# MicroRNA-126 protects against vascular injury by promoting homing and maintaining stemness of late outgrowth endothelial progenitor cells

**DOI:** 10.1186/s13287-020-1554-9

**Published:** 2020-01-21

**Authors:** Chong Zhe Pei, Bo Liu, Ye Ting Li, Lu Fang, Yi Zhang, Yi Gang Li, Shu Meng

**Affiliations:** 1 Department of Cardiology, Xinhua Hospital, School of Medicine, Shanghai Jiaotong University, Shanghai, China; 2 Haematopoiesis and Leukocyte Biology Laboratory, Baker Heart and Diabetes Research Institute, Melbourne, VIC Australia

**Keywords:** Endothelial progenitor cells, miR-126, Stemness maintenance, LOC homing, Diabetes

## Abstract

**Background:**

Endothelial progenitor cells (EPCs) contribute to reendothelialization and neovascularization and protect against vascular injury and ischemia of various organs. We have previously shown downregulation of microRNA (miR)-126 in EPCs from diabetic patients, which contributes to dysfunction of EPCs including impaired migratory ability. The aims of the present study were to examine (1) in vitro the effects of miR-126 on the homing and stemness of late outgrowth EPCs (LOCs), along with relevant signaling pathways, and (2) in vivo the effects of modulating LOCs by manipulating miR-126 expression on LOC homing and reendothelialization of injured arteries in GK rats (a non-obese diabetes model).

**Methods:**

Rat bone marrow-derived LOCs were transfected with miR-126 inhibitor or lentiviral vectors expressing miR-126. LOC migration was determined by transwell migration assay. CXCR4 expression was measured by real-time PCR, Western blotting, and confocal microscopy while related signaling pathway proteins were measured by Western Blotting. Stemness gene expression, and gene and protein expression and promoter activity of KLF-8 were also measured. LOCs transfected with lenti-miR-126 or miR-126 inhibitor were injected into GK rats with carotid artery injury, and then vascular reendothelialization and the extent of intimal hyperplasia were examined.

**Results:**

Lenti-miR-126 increased while miR-126 inhibitor decreased LOC migration and CXCR4 expression on LOCs. miR-126 positively regulated p-ERK, VEGF, p-Akt, and eNOS protein expression, and inhibitors of these proteins blocked miR-126-induced CXCR4 expression and also reduced LOC migration. Overexpression of miR-126 promoted while inhibition of miR-126 suppressed stemness gene expression in LOCs. miR-126 also inhibited gene and protein expression and promoter activity of KLF-8 while shRNA-mediated knockdown of KLF-8 increased stemness gene expression. Upregulation of stemness gene expression by miR-126 overexpression was completely abrogated by co-transfection of lenti-KLF-8 and lenti-miR-126 into LOCs. In GK rats, transplantation of LOCs overexpressing miR-126 enhanced LOC homing and reendothelialization and decreased intimal hyperplasia of injured arteries.

**Conclusion:**

Our results indicate that miR-126 protects against vascular injury by promoting CXCR4 expression and LOC homing via ERK/VEGF and Akt/eNOS signaling pathways and maintaining stemness via targeting KLF-8.

## Background

Endothelial progenitor cells (EPCs) are bone marrow-derived precursors of vascular endothelial cells and mobilize to injured endothelium or ischemic tissues where they participate in the repair of damaged endothelium and neovascularization of ischemic tissues [1–5]. Recent development in the characterization of human peripheral blood mononuclear cell-derived EPCs has identified two types of EPCs, named as early EPCs and late-outgrowth EPCs (LOCs) [6–9]. Rat bone marrow-derived EPCs were further classified into three subpopulations. Early EPCs appeared on days 3–6, while late-outgrowth and very late-outgrowth EPCs were defined as cells forming cobblestone colonies on days 9–14 and days 17–21, respectively [10]. While different subtypes of EPCs all contribute to endothelial repair of vascular injury, LOCs are able to differentiate into endothelial cells but early EPCs exert beneficial effects through their pro-angiogenic paracrine actions [11, 12]. Many factors, such as the number and migratory activity, correlate with circulating EPCs’ capacity of reendothelialization [13]. Diabetes are not only associated with reduced circulating EPC levels [14], but also associated with impairments of EPC mobilization and other functions [15–18]. Impaired vascular repair by malfunctioning EPCs in diabetes could play a pathologic role in increased cardiovascular risk associated with diabetes.

MicroRNAs (miRs), endogenous ~ 22 nt non-coding RNAs, are important regulators of EPC function post-translationally [19, 20]. MicroRNA-126 has been proved to play a critical role in vascular homeostasis [21, 22]. Our previous study reported that miR-126 expression was lower in peripheral blood-derived EPCs from diabetes, which contributes to functional impairments of diabetic EPCs [23]. Stromal cell-derived-factor 1α (SDF-1α)/chemokine receptor 4 (CXCR4) is a key interaction axis in regulating EPC homing [24]. Hamed et al. reported that the expression of CXCR4 was significantly reduced in EPCs from patients with diabetes or in EPCs treated with high glucose [25]. In addition to homing, stemness maintenance of EPCs is also essential for their role in tissue repair. A previous study showed that the first generation of primary MSCs had greater capacity than the fifth generation in repairing infarcted myocardium of mice [26], which was related to the difference in their maintenance of stemness [26]. Adrienne et al. found that miR-126 played a key role in the stemness maintenance of leukemia stem cells and that interference with miR-126 resulted in in vivo reduction of leukemia stem cells [27]. KLF family members play critical roles in vascular wall homeostasis [28]. The KLF family has been shown to play an important role in maintaining stemness [29, 30]. Although there is no correlation between KLF-8 and stemness  reported yet, KLF-8 plays an important role in cell proliferation [31] and epithelial-mesenchymal transition (EMT) process [32].

We thus speculated that the lower level of miR-126 in diabetic LOCs might impair migration via downregulating CXCR4 and decrease stemness of LOCs via regulating KLF-8, and thus decreasing endothelial repair ability. Therefore, the aims of the present study were to examine the effects of miR-126 on the homing and stemness maintenance of LOCs along with signaling pathways in vitro, and also to investigate in vivo whether modulating LOCs by overexpressing miR-126 enhanced LOC homing and reendothelialization of injured arteries in GK rats (a non-obese type II diabetes model).

## Materials and methods

### Isolation and identification of bone marrow-derived EPCs

Bone marrow from femurs of GFP-positive and normal Wistar rats (4–6 weeks, Shanghai Slac Laboratory Animal Co. Ltd.) was obtained by means of fine-needle aspiration. Bone marrow-derived mononuclear cells (BMMNCs) were isolated using Ficoll-Isopaque Plus (Sigma, USA) density gradient centrifugation. The cells were cultivated with EBM medium with EGM-2 MV SingleQuots (Lonza, Walkersville, MD, USA) at 37 °C in a 5% CO_2_ incubator. After 4 days, non-adherent cells were removed by washing with phosphate buffered saline (PBS). LOCs were defined as adherent cells on days 10–14 after culture as reported [10]. After 10 days of culture, the cells from normal Wistar rats were incubated with fluorescein isothiocyanate conjugated lectin from Ulex europeus agglutinin 1 (FITC-UEA-1) (Sigma, Germany), and 1,19-dioctadecyl-3,3,3939-tetramethylindocar-bocyanine perchlorate-labeled acetylated low-density lipoprotein (LDL-ac-Dil) (Sigma, Germany). Cells positive for both LDL-ac-Dil and UEA-1 were identified as LOCs. The purity of the LOCs was analyzed by flow cytometry after staining with antibodies, CD34, CD133, KDR, and CD31 (all from Bio Legend Inc., USA).

### Lentiviral construct, packaging, and infection

Expression plasmid for miR-126 was created using PCR amplification with rat genomic DNA as templates. The complete pri-miRNA sequence was used to generate the expression plasmid for miR-126, with the following primers: miR-126 F: 5′-TGACAGCACATTATTACTTTTGGTACGCG-3′, miR-126 R: 5′-TGACCACGCATTATTACTCACGGTAC-3′. The PCR products of miR-126 were cloned into the lentiviral expression plasmid pCDH-CMV-MCS-EF1-Puro (System Biosciences). The construct of miR-126 was confirmed by sequencing. The plasmid DNA was co-transfected into HEK293 T cells with pCDH-126, psPAX2, and pMD packaging construct using lipo 2000 (Invitrogen) according to the manufacturer’s protocol. Medium was refreshed after 6 h, and lentiviral supernatant was collected 48 h later. LOCs were seeded into six-well plates (5 × 10^5^ cells/well) and infected with different lentiviral vectors at a MOI of 10. To inhibit miR-126 expression, a miR-126 inhibitor and its negative control (scramble) were transfected into LOCs. The oligonucleotide sequences of miR-126 inhibitor were 5′-CGCAUUAUUACUCACGGUACGA-3′ and sequences of negative control were 5′-CAGUACUUUUGUGUAGUACGA-3′. Both were synthesized by the System Biosciences Co. Ltd. (New Jersey, USA). The transfected mixtures were configured according to the instructions of the Lipofectamine 2000 kit (Invitrogen Inc., USA). Briefly, 5 μL inhibitor (dissolved in PBS) and 250 μL optimum were mixed, so as 5 μL lipo 2000 and 250 μL optimum. Twenty minutes later, two types of mixtures were mixed together. Then the final mixture was added into six-well plates (5 × 10^5^ cells/well) and cells were transfected with miR-126 inhibitor and negative control (the final dose was 100 nM). The transfection efficiency was confirmed by real-time PCR.

### Pretreatments with signaling pathway inhibitors

PD98059 (an ERK inhibitor, Selleck, USA), GW654652 (a vascular endothelial growth factor (VEGF) inhibitor, TargetMol, USA), PX-316 (an Akt inhibitor, BOC Science, USA), and 7-nitroindazole (an endothelial nitric oxide synthase (eNOS) inhibitor, Sigma, USA) were all dissolved in DMSO. All inhibitors (10 μmol) were added 1 h prior to infection with lenti-miR-126 for 24 h.

### Manipulation of KLF8 and reporter gene assay

Short hairpin (sh) RNA against KLF8 and negative control shRNA were cloned into the pLL3.7 lentiviral vector. The KLF8 coding sequence was amplified and cloned into the pWPI-eGFP lentiviral vector to generate the KLF8 overexpression vector, while the empty vector served as a negative control. The shRNA sequence targeting KLF8 was 5′-CAGAAGAACTTTTGGCTAG-3′, and the control shRNA sequence was 5′-CAGTCGCGTTTGCGACTGG-3′. Luciferase reporter experiments were performed as previously described [33]. The 3′UTR of the KLF-8 gene was amplified by PCR from human genomic DNA and cloned into the psiCHECKTM-2 vector (Promega) between the Not1 and Sgf1 sites. The construct with mutated targeting fragment at the 3′UTR of KLF-8 without the putative miR-126 binding sequence was used as a mutated control. LOCs were seeded into 24-well plates (1 × 10^4^ cells/well) by using lipofectamine 2000 according to the manufacturer’s protocol. Cells treated with 10 nM miR-126 inhibitor or negative control, or with 10 nM lenti-miR-126 or lenti-vector were transfected with 0.1 mg of the p-KLF-8 UTR firefly luciferase report vector and 0.02 mg pRL-TK (Promega) for normalization of transfection. After 48 h, cells were washed and lysed with passive lysis buffer, and firefly luciferase activity was determined using the dual-luciferase reporter assay system and a luminometer (Promega). The relative reporter activity was obtained by normalizing the firefly luciferase activity against the internal control luciferase activity.

### RNA isolation and real-time PCR

Total RNA was extracted from LOCs or carotid arteries using Trizol reagent (Invitrogen, USA) according to the manufacturer’s instruction. Extracted RNA was further purified (Small RNA Gel Extraction Kit, TaKaRa Bio Inc. Japan) and reverse-transcribed by using the TIANGEN miRNA Reverse Transcription Kit (TIAN GEN BIOTECH, China). Specific primers for different genes are described in Table 1. The PCR reaction was performed as follows: stage 1, 94 °C for 2 min, stage 2, 94 °C for 20 s, and 60 °C for 34 s. Stage 2 was repeated for 40 cycles. Real-time PCR was performed using SYBR&ROX PCR master mix (TIAN GEN BIOTECH, China) with Applied Biosystems ABI7500 Real-time PCR System. GAPDH was used as an endogenous control. All samples were normalized to internal controls, and the relative expression level was calculated using the 2^−ΔΔCt^ analysis method.

**Table 1:** Sequences of real-time PCR primers used in this study

Name	Sequence (5′–3′)
miR-126	F: 5′-TATAAGATCTGACGATAGGTGGGTTCCCGAGAACT-3′
	R: 5′-ATATGAATTCTCTCAGGGCTATGCCGCCTAAGTAC-3′
CXCR4	F: 5′-GTCAACCTCTACAGCAGCGT-3′
	R: 5′-CTATCGGGGTAAAGGCGGTC-3′
GAPDH	F: 5′-AGACAGCCGCATCTTCTTGT-3′
	R: 5′-CTTGCCGTGGGTAGAGTCAT-3′
KLF-8	F: 5′-TCTGCAGGGACTACAGCAAG-3′
	R: 5′-TCACATTGGTGA ATCCGTCT-3′
Oct-4	F: 5′-CGCCCGCATACGAGTTC-3′
	R: 5′-CTTCTCCAACTTCACGGCATT-3′
Sox-2	F: 5′-CCAGCGCATGGACAGCTA-3′
	R: 5′-GCTGCTCCTGCATCATGCT-3′
Nanog	F: 5′- CGTTCCCAGAATTCGATGCTT-3′
	R: 5′- TTTTCAGAAATCCCTTCCCTCG-3′
Rex-1	F: 5′- AGATGGCTTCCCTGACGGATA-3′
	R: 5′- CCTCCAAGCTTTCGAAGGATTT-3′

### Migration assay

Migration assay was done as previously described [34]. LOCs (1 × 10^6^/mL) were resuspended in serum-free EBM medium, and 1 × 10^5^ cells were loaded into the upper transwell chambers (Corning, Corning incorporated Life Science, USA), and the lower chambers were filled with EBM medium supplemented with different concentrations (0, 25, 50, 100, or 200 ng/mL) of SDF-1α (Peprotech Asia, USA). The assays were conducted over a 24-h incubation period at 37 °C in an incubator equilibrated with 5% CO_2_. The membrane was then washed gently with PBS, and non-migrated cells were removed with cotton balls from the upper side of the membrane while migrated cells were fixed with 4% paraformaldehyde. The membrane was then stained by using 0.1% crystal violet solution for 30 min. Migrated LOCs were counted under a microscope (CX31, Olympus Japan) in four random high-power fields (× 100) in each membrane. All groups were performed in triplicate.

### Protein isolation and Western blotting

Protein isolation and Western blotting were performed as previously described [35]. Total cellular protein was extracted in RIPA lysis buffer (Beyotime, China) supplemented with 1% PSMF (Beyotime, China) and 1% phosphatase inhibitor (Beyotime, China). Protein was quantified using the bichoninic acid assay (BCA; Beyotime, China) according to the manufacturer’s instructions. Equal amounts of protein (30 μg) were separated through a 10% sodium dodecyl sulfate- polyacrylamide gel electrophoresis (SDS-PAGE) and then transferred to a PVDF membrane. Membranes were blocked in 5% milk-Tris-buffered saline with Tween 20 (TBST), followed by overnight incubation with primary antibodies for phosphor (p)-ERK, ERK, VEGF, p-Akt, Akt, eNOS, CXCR4, KLF-8, and GAPDH (dilution 1:1000, Abcam, USA) at 4 °C. The membranes were washed with TBST and then incubated with anti-rabbit or anti-mouse secondary antibodies (dilution 1:1000, Beyotime, China). All signals were detected by the Molecular Imager ChemiDocTM XRS+ System (BIO-RAD, USA), and data were normalized by GAPDH levels (p-ERK and p-Akt were normalized by total ERK and total Akt, respectively).

### Confocal microscopy

For confocal microscopy, LOCs were first transfected with miR-126 inhibitor or negative control, or lenti-miR-126 or lenti-vector. Following blockade with 1% bovine serum albumin (BSA)/PBS for 1 h, cells were then incubated for 1 h at room temperature with rabbit anti-rat CXCR4 antibody (1:50) (Abcam, USA) rabbit anti-rat Oct-4 antibody (1:50) (Abcam, USA), rabbit anti-rat Sox-2 antibody (1:50) (Abcam, USA), rabbit anti-rat Nanong antibody (1:50) (Abcam, USA), and rabbit anti-rat Rex-1 antibody (1:50) (Abcam, USA). After being washed with PBS containing 0.1% Tween-20, the samples were incubated with secondary antibodies (Alexa Fluor 647 mouse-anti-rabbit IgG at 1:200, Invitrogen) for 2 h at room temperature. Following fixation, the cells were mounted in ProLong Gold antifade reagent with DAPI (eBioscience, USA) to stain the nucleus. Cells were then examined on a LSM510 confocal laser scanning microscope (Carl Zeiss Inc., Minnesota, USA).

### Rat carotid artery injury model and LOC transfer

GK rats (12–14 weeks) weighing 250 to 300 g were obtained from Shanghai SLAC Laboratory Animal Co. Ltd. All animal experimental methods were approved by the Ethics Committee of Shanghai Jiaotong University School of Medicine. Carotid artery balloon injury was performed in GK rats as previously described [36, 37]. GK rats were anesthetized by an intraperitoneal injection of 3% pentobarbital (0.1 mL/100 g). A 1.25 × 15 mm balloon catheter (Abbott, USA) was introduced into the left external carotid artery and then advanced for at least 4 mm towards the aorta. The balloon was inflated with 0.02 mL of PBS and then withdrawn through the common carotid artery to the carotid bifurcation, with constant rotation during denudation of the endothelium. This procedure was repeated three times. Two hours after injury, LOCs transfected with miR-126 inhibitor or lenti-miR-126 or the respective control were injected into the tail vein with a volume of 100 μL (containing 1 × 10^6^ cells), while the control group was injected with 100 μL PBS. In sham operation group, animals were only anesthesized and had the left external carotid artery exposed. 16 GK rats were used for each group.

### Quantification of FBG, AGEs, IL-6, and TNF-α levels

Blood was taken at baseline  before operation and 28th day after the operation. Serum samples were obtained by centrifuging the blood at 1600 g for 15 min at room temperature within 30 min of tail vein puncture, and aliquots were stored immediately at − 80 °C for future analysis. Fasting blood glucose (FBG) was measured by the hexokinase method. Plasma samples were analyzed for advanced glycation end products (AGEs), interleukin (IL)-6, and tumor-necrosis factor (TNF)-α with ELISA kits (eBioscience, San Diego, CA, USA) according to the manufacturer’s instructions. All samples were assayed in duplicate, and values were analyzed according to standard curves. The lower detection limit for these three assays is 0.005 ng/mL. Blood samples used for this analysis were restricted to a single freeze–thaw cycle.

### Histological analysis and immunohistochemistry staining

Histological analysis and immunohistochemistry staining were performed as previously described [38]. OCT-embedded common carotid arteries were cut systematically in serial 5-μm cross sections using a cryotome (Leica CM3050S, Leica Microsystems, Germany). Analysis was carried out in the injured left common carotid artery, whereas the contralateral served as a control. For morphometric analysis, sections were stained with hematoxylin and eosin (HE). For immunofluorescence analysis, sections were stained with a rabbit anti-rat CD31 antibody (1:250, Abcam, USA) and visualized using an Alexa Fluor 647 mouse-anti-rabbit IgG secondary antibody (1:500, eBioscience). For immunohistochemistry analysis, sections were stained with a rabbit anti-rat CD31 antibody (1:150, Abcam, USA) and visualized using a mouse-anti-rabbit secondary antibody (1:500, Sigma). Negative controls using IgG controls matching in species and concentration were run in parallel. Pictures were taken with a microscope (Leica CM3050S, Leica Microsystems, Germany) and a digital camera (DFC 320, Leica Microsystems) at ×100 magnification. Morphometric analysis was performed per sample followed by computer-assisted morphometric analysis (ImageJ, NIH, USA). Subsequent morphometric analyses were performed in a double-blinded manner. Four animals per group and 3 samples per animal were analyzed.

### Evans blue staining

Seven days after carotid injury, reendothelialization was evaluated by staining of the denuded area with 50 μL of 2% Evans blue solution (Sigma-Aldrich, Germany) via injection into the left ventricle of the heart. Rats were killed 15 min after injection of Evans blue and then perfused with 4% paraformaldehyde solution (PFA) via the left ventricle. The reendothelialized area was calculated as difference between the blue-stained area and the initially injured area by computer-assisted morphometric analysis (ImageJ, NIH, Bethesda, MD, USA) and presented as percentage of reendothelialization (defined as area not stained with Evans blue/total injured surface area). All vessels were taken starting from bifurcation point towards arch of aorta with a length of 10 mm and examined from one side (left side). Four animals per group were analyzed.

### Statistical analysis

Each experiment was performed at least three times, and all values were represented as mean ± SD. Data were compared using two-tailed Student’s *t* test for two independent samples or one-way ANOVA and a multiple comparison post hoc test for more than two groups. *p* < 0.05 was considered statistically significant. SPSS 20.0 software was used for statistical calculations.

## Results

### In vitro studies

#### Characterization and identification of LOCs

BMMNCs were isolated and plated on fibronectin-coated culture plates. Ten days after plating, adherent LOCs with spindle shape-formed clones (Additional file 1: Figure S1A,1B). Isolated LOCs were GFP positive from GFP positive Wistar rats (Green) (Additional file 1: Figure S1C). Most LOCs were shown to simultaneously endocytose DiI-ac-LDL (red) (Additional file 1: Figure S1D) and bind to fluorescein isothiocyanate UEA-1 (lectin, green) from normal Wistar rats (Additional file 1: Figure S1E). FACS analysis showed high expressions of CD34 (93.6%), CD133 (95.4%), and KDR (91.1%) and low expression of CD31 (10.1%) on the surface of LOCs (Additional file 1: Figure S1G) after 14 days of culture.

#### miR-126 promo0074ed LOC migration

**Fig. 1:**
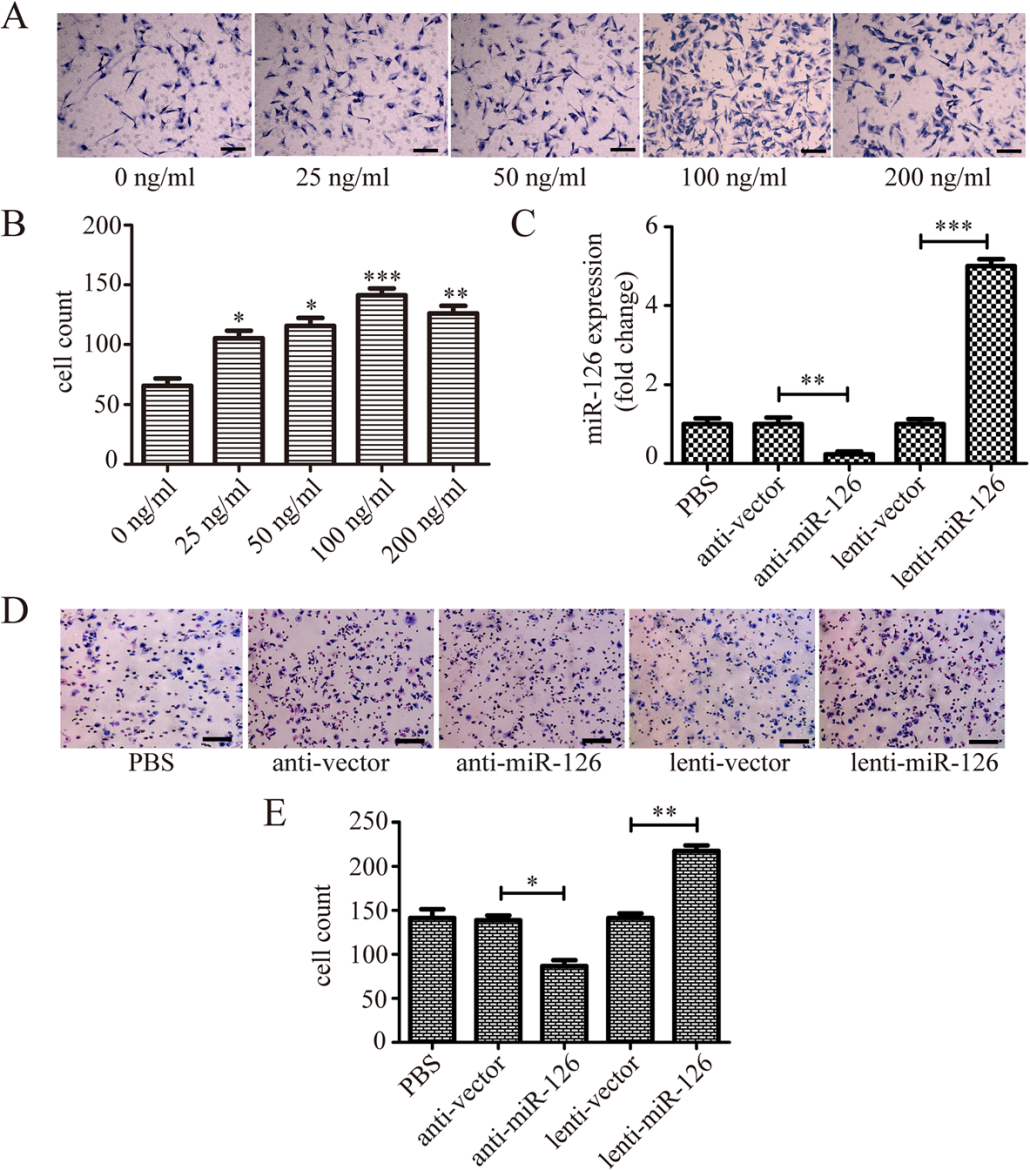
The effect of miR-126 on LOC migration. **a**, **b** The migration of LOCs in response to different concentrations of SDF-1α (25, 50, 100, 200 ng/mL). **c** The transfection efficiency of miR-126 was confirmed by real-time PCR. **d**, **e** The effect of miR-126 on LOC migration in the presence of 100 ng/mL of SDF-1α. **d** Crystal violet staining was performed to determine the number of migrated cells. **e** Cell counts per high-power field were analyzed by ImageJ. Data are presented as mean ± SD. *P < 0.05, **P < 0.01, and ***P < 0.001, vs respective control group; *n* ≥ 3. Scale bar = 100 μm

First, we tested the effects of different concentrations of SDF-1α (25, 50, 100, or 200 ng/mL) on LOC migration (Fig. 1a, b) and selected 100 ng/mL of SDF-1α for subsequent experiments since it was the optimal concentration. We next confirmed alterations of miR-126 expression in LOCs by transfection with lenti-miR-126 or miR-126 inhibitor (Fig. 1c). Then, we found that LOC migration in the presence of 100 ng/mL of SDF-1α was dramatically inhibited by miR-126 inhibitor, while augmented by lenti-miR-126 (Fig. 1d, e).

#### miR-126 regulated CXCR4 expression on LOCs

**Fig. 2:**
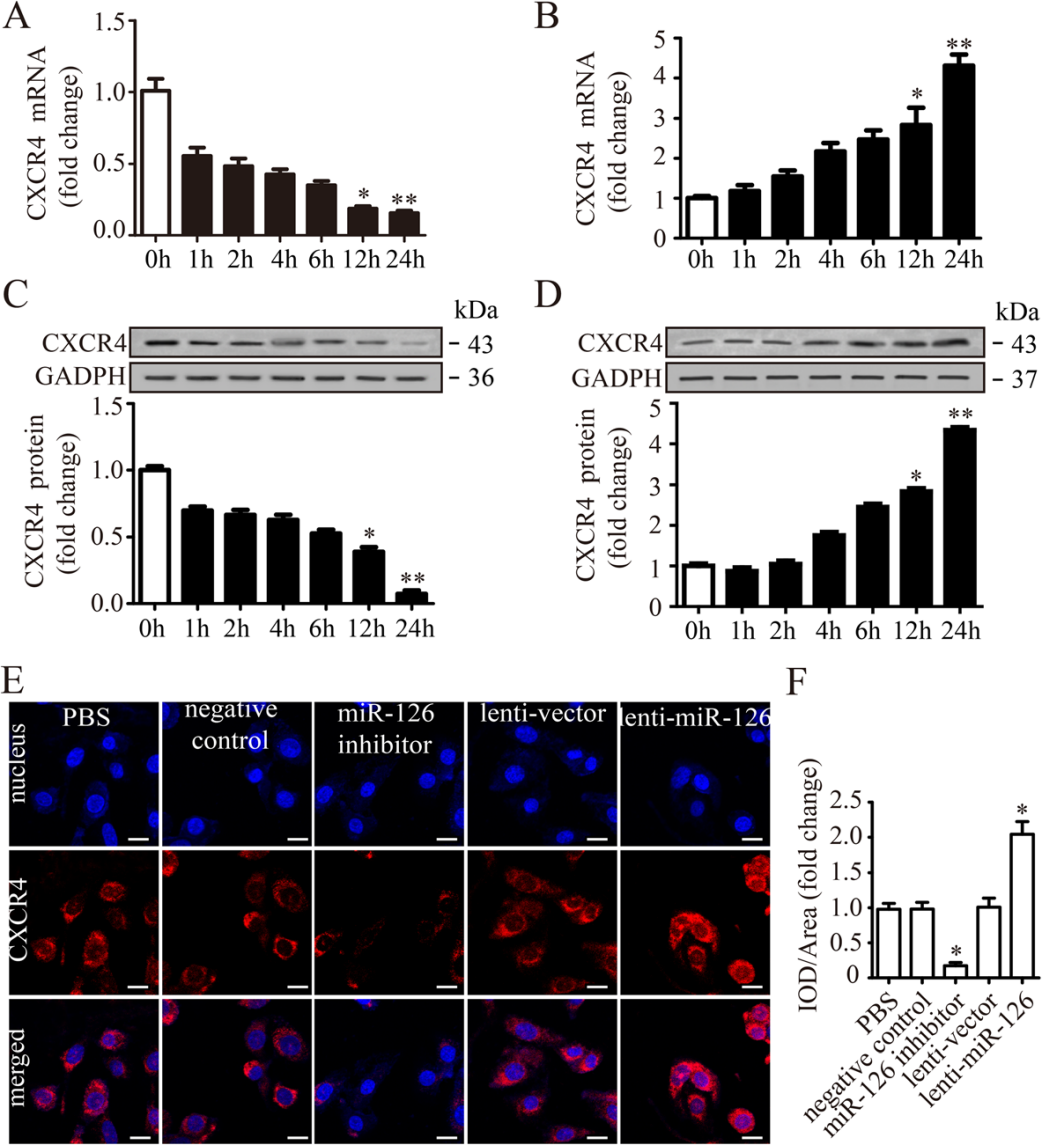
The effect of miR-126 on CXCR4 expression on LOCs. Relative gene expression was determined for CXCR4 by real-time PCR at 0 h, 1 h, 2 h, 4 h, 6 h, 12 h, and 24 h after transfection with miR-126 inhibitor (**a**) or lenti–miR-126 (**b**). Western blots show the protein expression of CXCR4 at 0 h, 1 h, 2 h, 4 h, 6 h, 12 h, and 24 h after transfection with miR-126 inhibitor (**c**) or lenti–miR-126 (**d**). A laser scanning confocal microscope was used to confirm CXCR4 expression on the cell membrane of LOCs in different groups (**e**). Mean fluorescence intensity was calculated by average area intensities using image J (**f**). Data are presented as mean ± SD. *P < 0.05, **P < 0.01 vs respective control group; *n* ≥ 3. Scale bar = 100 μm

Inhibition of miR-126 (Fig. 2a, c) downregulated while overexpression of miR-126 (Fig. 2b, d) upregulated the gene and protein expression of CXCR4 in a time-dependent manner, measured by real-time PCR (Fig. 2a, b) and Western blotting (Fig. 2c, d), respectively. Laser scanning confocal microscopy also confirmed that lenti-miR-126 increased while miR-126 inhibitor decreased CXCR4 expression on the cell surface of LOCs, compared with their respective control group (Fig. 2e, f).

#### The regulation of CXCR4 expression on LOCs by miR-126 mediated via Akt/eNOs and ERK/VEGF signaling pathways

**Fig. 3:**
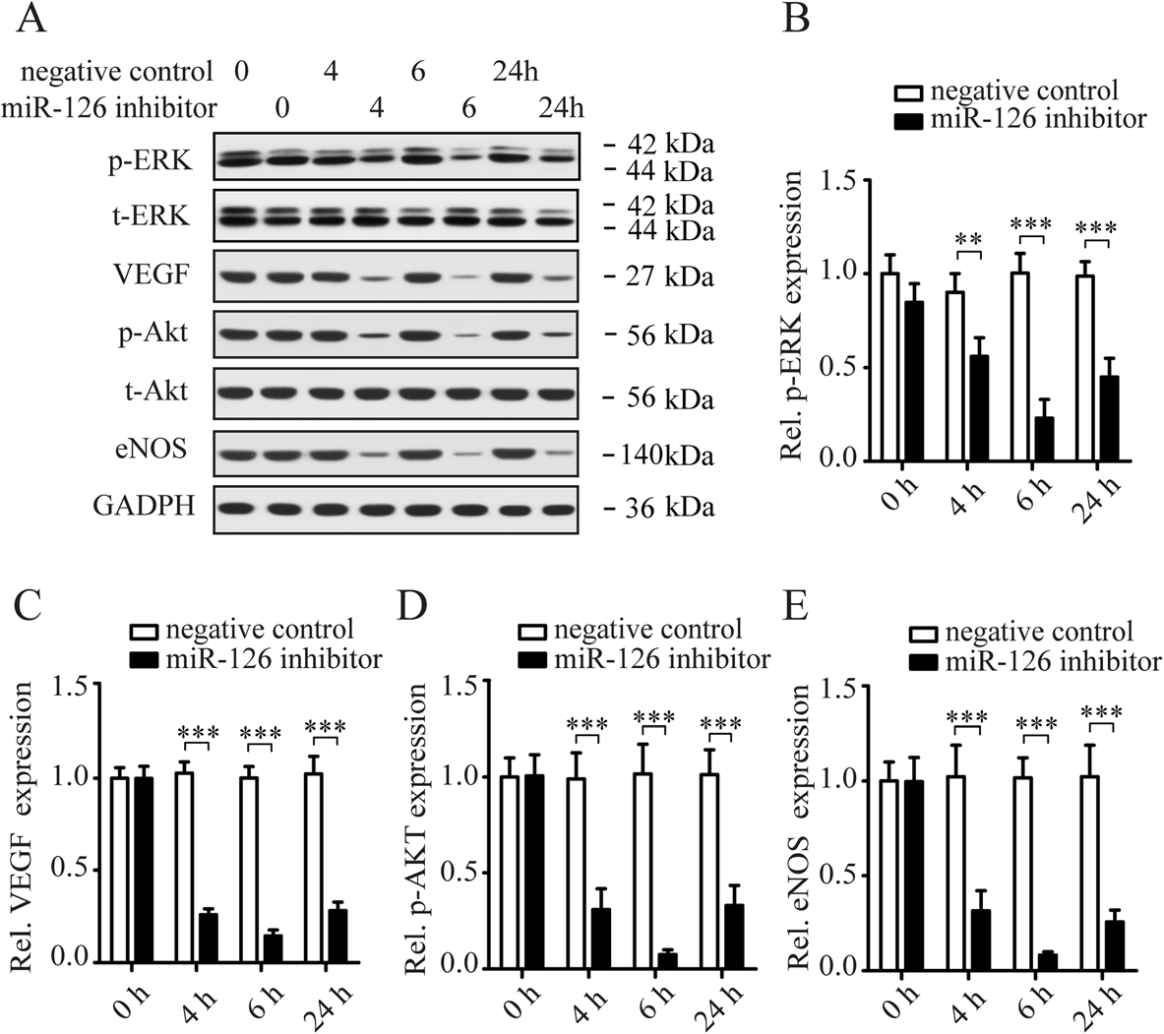
Inhibition of miR-126 downregulates protein expression of Akt/eNOS and ERK/VEGF signaling pathways. Western blotting shows immunochemistry signals of phosphorylated-ERK (p-ERK), ERK, VEGF, p-Akt, Akt, and eNOS in LOCs transfected with negative control or miR-126 inhibitor (**a**). Quantification of p-ERK (**b**), VEGF (**c**), p-Akt (**d**), and eNOS (**e**) bands. GADPH expression was used for protein level normalization for VEGF and eNOS. Data are presented as mean ± SD. **P < 0.01, ***P < 0.001 vs. respective control group; *n* ≥ 3

**Fig. 4:**
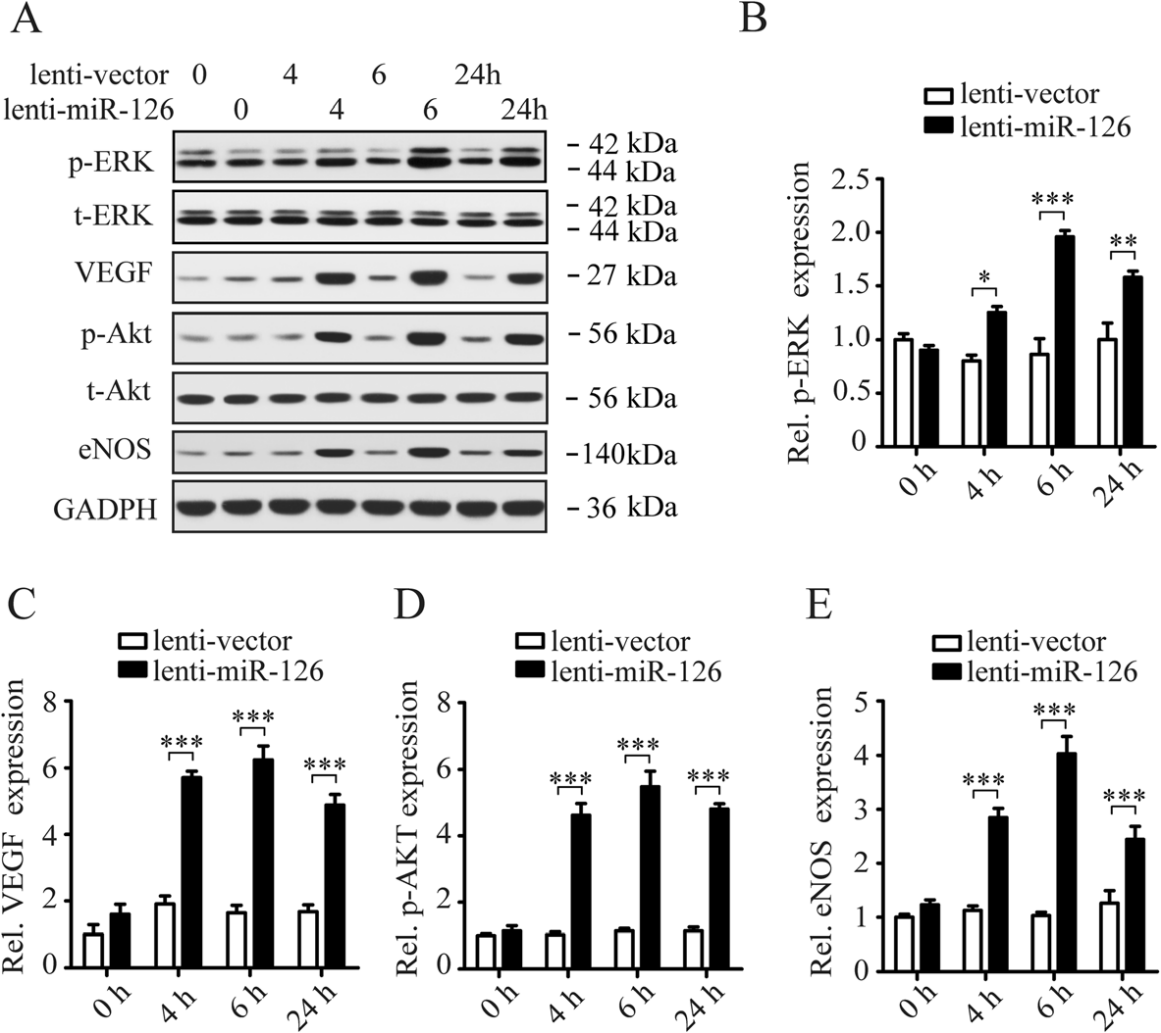
Overexpression of miR-126 activates P13K/Akt/eNOS and ERK/VEGF signaling pathways in LOCs. Western blotting shows immunochemistry signals of ERK, phosphorylated-ERK (p-ERK), VEGF, Akt, p-Akt, and eNOS in LOCs infected with lenti-vector or lenti-miR-126 (**a**). Quantification of p-ERK (**b**), VEGF (**c**), p-Akt (**d**), and eNOS (**e**) bands. GADPH expression was used for protein level normalization for VEGF and eNOS. Data are presented as mean ± SD. *P < 0.05, **P < 0.01, ***P < 0.001 vs. respective control group; *n* ≥ 3

**Fig. 5:**
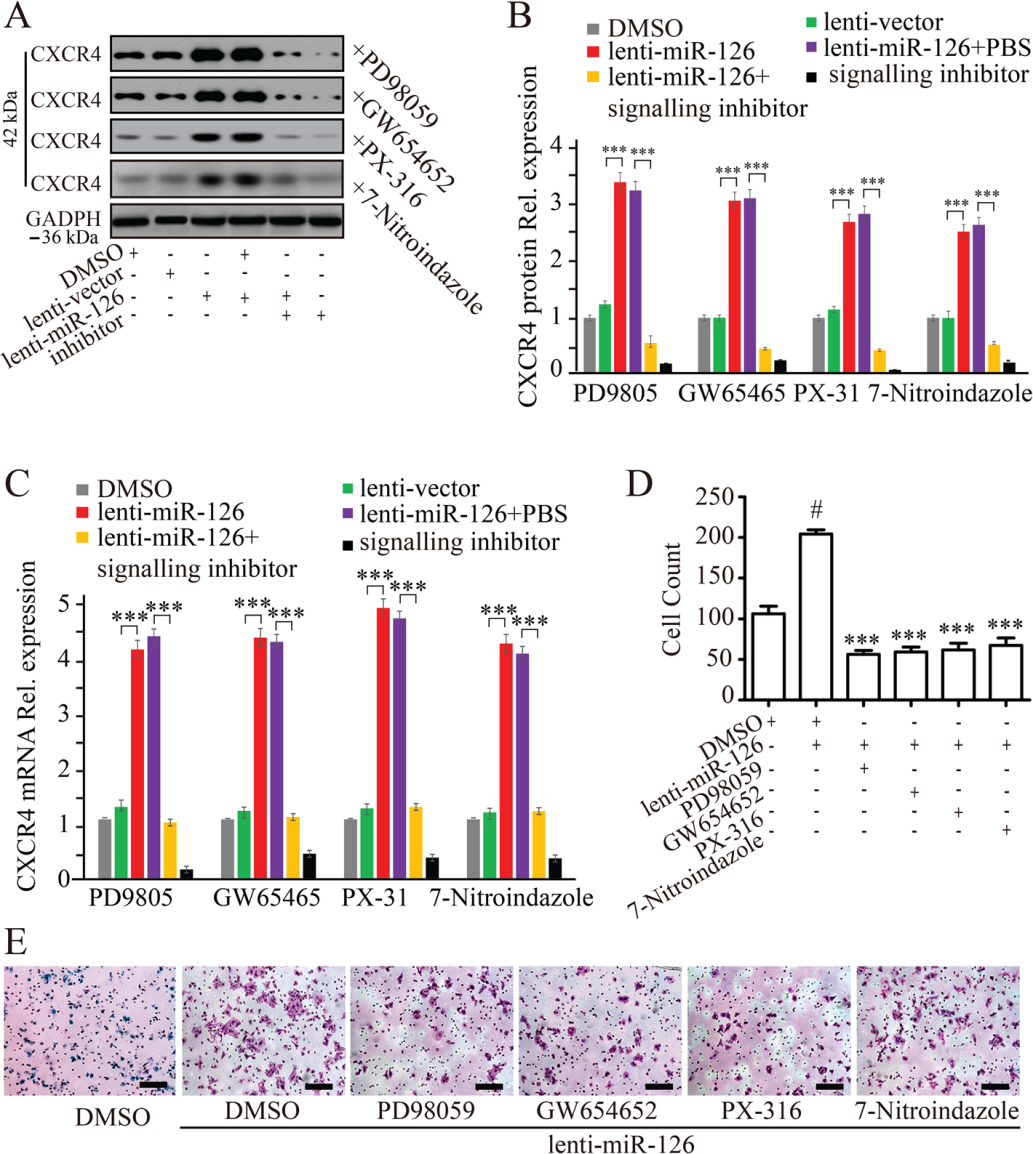
Inhibitors of AKT/eNOS and ERK/VEGF pathways regulate CXCR4 expression and LOC migration. Protein (**a**, **b**) and mRNA (**c**) expression of CXCR4 measured by Western blotting and real-time PCR on LOCs pretreated with PD98059 (10 μM), GW644652 (10 μM), PX-316 (10 μM), or 7-nitroindazole (10 μM) for 1 h before infection with lenti-miR-126. Representative western blots of CXCR4 (**a**). Quantification of CXCR4 bands on LOCs pretreated with PD98059, GW644652, PX-316, or 7-nitroindazole (**b**). GADPH expression was used for protein level normalization. mRNA expression of CXCR4 (**c**). miR-126 induced LOC migration in response to SDF-1α (100 ng/mL) was dramatically inhibited by PD98059, GW644652, PX-316, or 7-nitroindazole (**d** cell count, **e** crystal violet staining). Data are presented as mean ± SD. ***P < 0.001 vs. lenti-miR-126 group; #*P* < 0.05 vs. DMSO group. *n* ≥ 3. Scale bar = 100 μm

We later examined the involvement of signaling pathways in regulating CXCR4 on LOCs by miR-126. Our previous study reported that the effect of miR-126 on regulating EPC function was mediated by ERK/VEGF and AKT/eNOS signal pathways [23]. So, we focused on AKT/eNOS and ERK/VEGF pathways since CXCR4 is an important mediator of EPC migration. First, we examined the regulation of signaling pathways in LOCs by miR-126. LOCs were transfected with miR-126 inhibitor or lenti-miR-126, and signal pathway proteins (p-ERK, t-ERK, VEGF, p-Akt, t-Akt, and eNOS) were examined by Western blotting at 0, 4, 6, and 24 h after transfection (Figs. 3a and 4a). We found that expression of p-ERK/t-ERK, VEGF, p-Akt/t-Akt, and eNOS were downregulated in miR-126 inhibitor-transfected LOCs (Fig. 3b–e) while upregulated in lenti-miR-126 infected LOCs (Fig. 4b–e). Then, we examined whether blockade of these signaling pathway proteins with specific inhibitors mediated the regulation of CXCR4 expression by miR-126. LOCs were pretreated with PD98059 (ERK inhibitor), or GW654652 (VEGF inhibitor), or PX-316 (Akt inhibitor), or 7-nitroindazole (eNOS inhibitor) for 1 h before being infected with lenti-miR-126 for 24 h. CXCR4 protein (Fig. 5a, b) and mRNA (Fig. 5c) expression was downregulated by inhibitors of ERK, VEGF, Akt, and eNOS, indicating that these signaling pathway proteins mediate the regulation of CXCR4 by miR-126. These inhibitors also reduced LOC migration induced by miR-126 overexpression in the presence of SDF-1 (Fig. 5d, e), suggesting that Akt/eNOS and ERK/VEGF-dependent signaling pathways mediate the effect of miR-126 on promoting EPC migration.

#### miR-126 contributed to stemness of LOCs

**Fig. 6:**
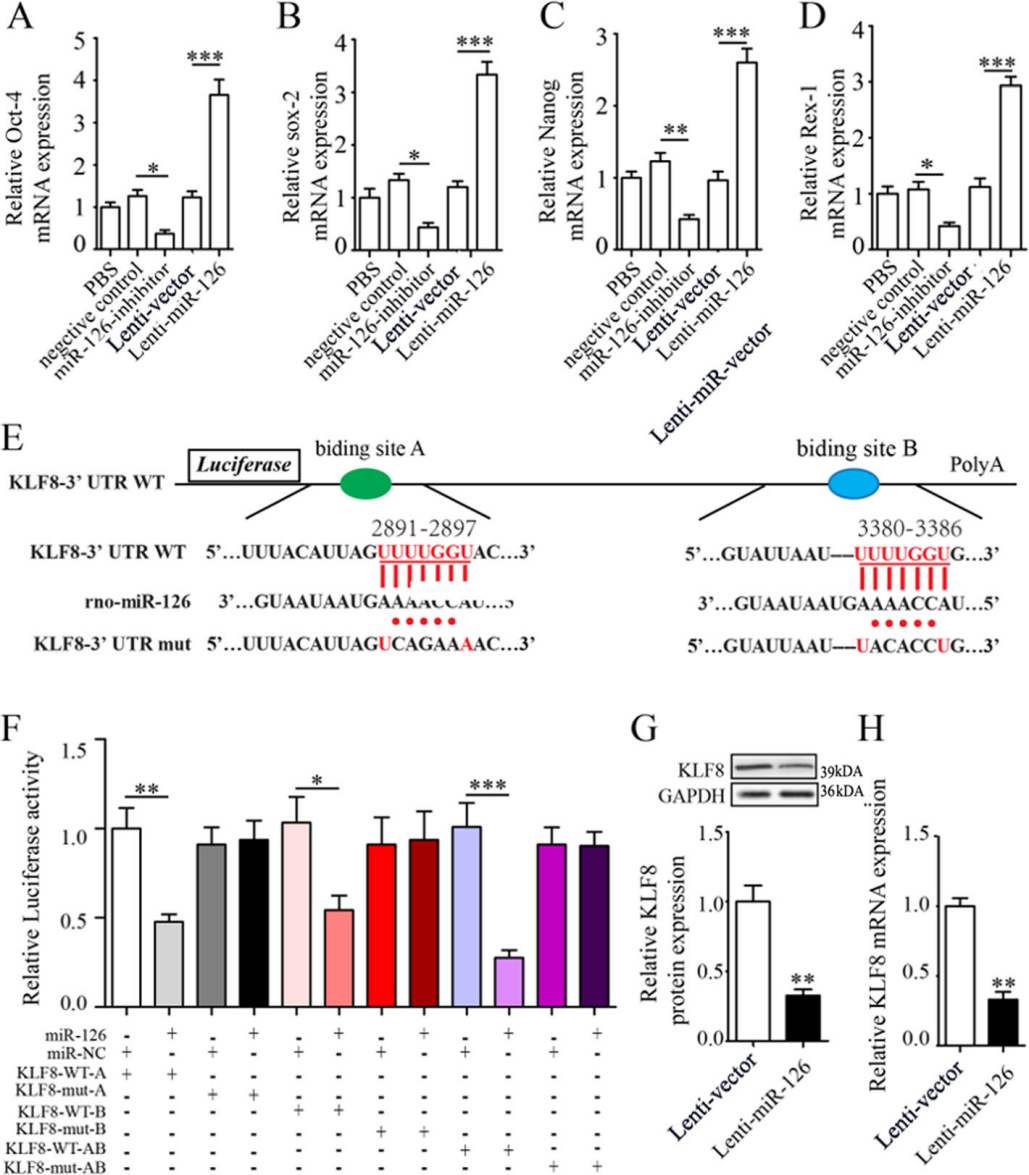
miR-126 contributes to LOC stemness. Relative gene expression was determined for Oct-4 (**a**), sox-2 (**b**), Nanog (**c**), and Rex-1 (**d**) by real-time PCR after transfection with miR-126 inhibitor or lenti–miR-126. KLF-8 mRNA wide-type (KLF-8 WT) and the mutated-type (KLF-8  mut) in the miR-126 binding sites were shown (**e**). Luciferase activity of LOCs co-transfected with lenti-miR-126 or lenti-miR-NC and luciferase reporters containing KLF-8 WT or KLF-8 mut transcript were determined by dual-luciferase reporter assays (**f**). KLF-8 protein (**g**) and mRNA (**h**) expression in LOCs transfected with lenti-miR-126 or lenti-miR-NC. Data are presented as mean ± SD. *P < 0.05, **P < 0.01, and ***P < 0.001, vs respective control group; *n* ≥ 3

We then found that manipulation of miR-126 altered gene and protein expression of stemness markers (Oct-4, sox-2, Nanog, and Rex-1) in LOCs as shown that anti-miR-126 significantly reduced while lenti-miR-126 significantly increased stemness gene expression (Fig. 6a, b) and protein expression (Additional file 3: Figure S3).

#### miR-126 modulated stemness of LOCs by targeting KLF-8

**Fig. 7:**
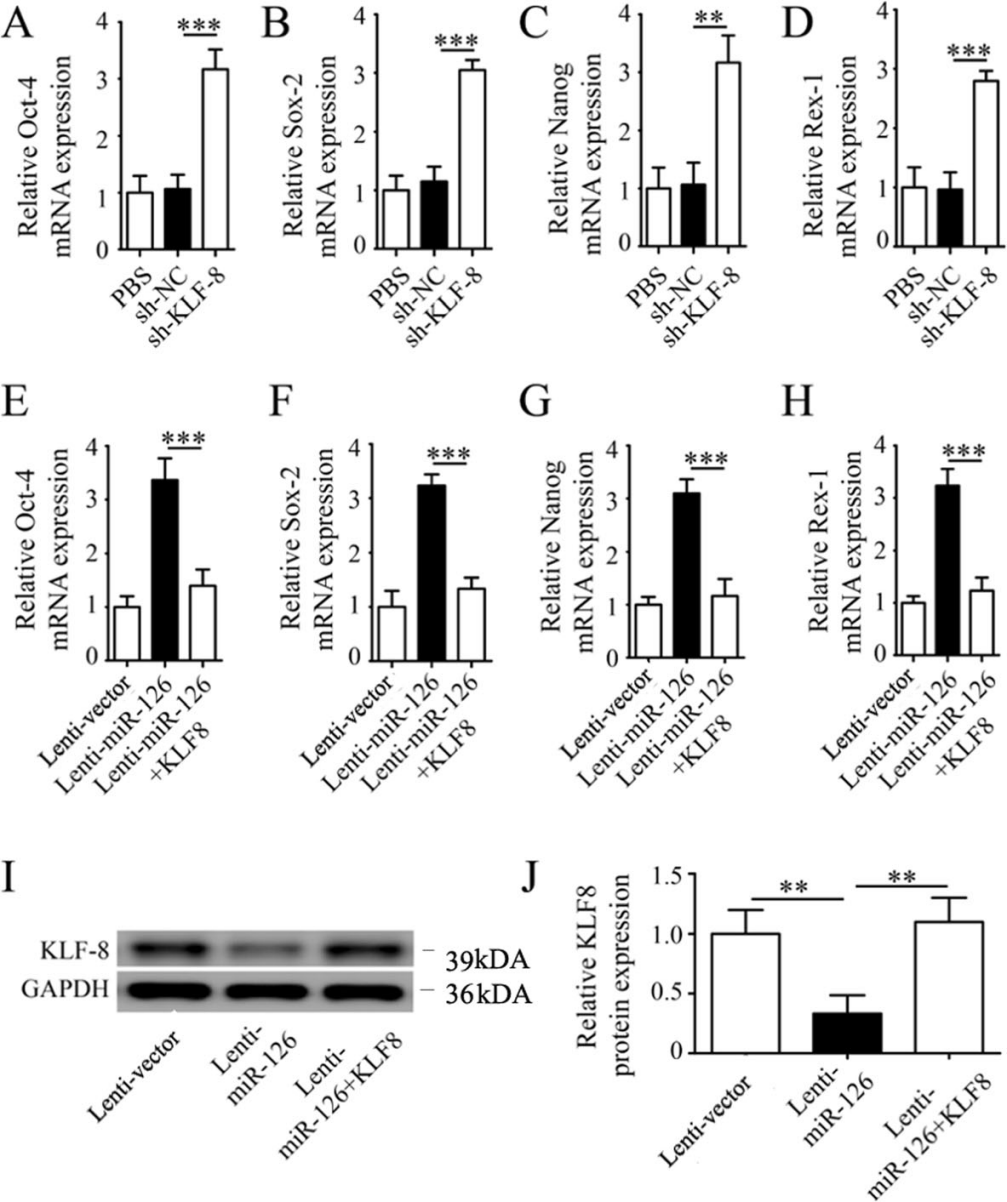
miR-126 modulates LOC stemness by targeting KLF-8. Relative gene expression was determined for Oct-4 (**a**), sox-2 (**b**), Nanog (**c**), and Rex-1 (**d**) by real-time PCR after transfection with sh-KLF-8 or sh-NC. Relative gene expression was determined for Oct-4 (**e**), sox-2 (**f**), Nanog (**g**), and Rex-1 (**h**) by real-time PCR after transfected with lenti-miR-126, lenti-miR-NC and co-transfected with lenti-miR-126 and lenti-KLF-8. KLF-8 protein (**i**, **j**) expression in LOCs transfected with lenti-miR-126 and lenti-miR-NC and co-transfected with lenti-miR-126 and lenti-KLF-8. Data are presented as mean ± SD. **P < 0.01 and ***P < 0.001, vs. respective control group; *n* ≥ 3

Since KLFs regulate stemness [29, 30], we next explored whether KLF-8 was involved in the role of miR-126 in maintaining stemness of LOCs. First, a dual luciferase reporter gene assay was performed to examine whether KLF-8 is a direct target of miR-126. Overexpression of miR-126 inhibited luciferase reporter activity with a wild-type sequence for KLF-8 (Fig. 6e, f); however, this effect was not observed for the mutants (Fig. 6e, f), confirming that KLF-8 is a direct target of miR-126 in LOCs. We also observed that miR-126 negatively regulated KLF-8 mRNA and proteins (Fig. 6g, h). Then, we explored whether KLF-8 mediated the effect of miR-126 on stemness of LOCs. We found that shRNA-mediated knockdown of KLF-8 increased the stemness gene expression profiles (Fig. 7a–d) and that upregulation of stemness gene expression profiles by miR-126 overexpression was completely abrogated by co-transfection of lenti-KLF-8 and lenti-miR-126 into LOCs (Figs. 7e–h). In addition, we confirmed that coinfection of KLF-8 and lenti-miR-126 offset the reduction of protein expression levels of KLF-8 by lenti-miR-126 (Fig. 7i, j). We further examined whether KLF-8 also mediates the migration of LOCs. We did not find that overexpression of KLF-8 affected the migration of lenti-miR-126-infected LOCs (Additional file 2: Figure S2).

### In vivo studies

#### Body weight, AGEs, IL-6, and TNF-α levels in GK rats

There were no differences in body weight, fasting blood glucose, AGEs, IL-6, and TNF-α among different groups at baseline before operation (data not shown). At the time of sacrifice at 28 days after operation, there were no differences in body weight, FBG, and AGEs levels among all groups (Table 2). IL-6 and TNF-α levels were lower (but not significant) in lenti-miR-126-infected LOC groups, compared with either lenti-vector group or PBS control group (Table 2).

**Table 2:** Body weight, AGEs, IL-6, and TNF-α levels in GK rats. Data are shown as means ± SD

	Weight (g)	FBG (mmol/L)	AGEs (mg/L)	IL-6 (pg/L)	TNF-α (pg/L)
PBS control	280 ± 15.0	14.12 ± 2.1	51.11 ± 3.2	9.70 ± 2.0	12.74 ± 1.8
Negative control transfected LOC	270 ± 12.3	13.79 ± 1.9	53.37 ± 2.7	9.35 ± 3.1	10.93 ± 2.1
miR-126 inhibitor transfected LOC	282 ± 14.2	13.64 ± 2.0	51.87 ± 3.1	9.01 ± 3.5	11.73 ± 2.3
Lenti-vector infected LOC	275 ± 15.8	14.88 ± 2.2	52.79 ± 3.3	8.78 ± 2.6	11.56 ± 2.5
Lenti-miR-126 infected LOC	284 ± 13.8	14.73 ± 1.8	50.88 ± 2.8	6.72 ± 2.5	8.39 ± 1.7
Sham	279 ± 17.2	15.16 ± 2.4	49.50 ± 4.3	9.42 ± 2.9	11.88 ± 2.4

#### Lenti-miR-126-infected LOCs promoted vascular reendothelialization after carotid vascular injury

**Fig. 8:**
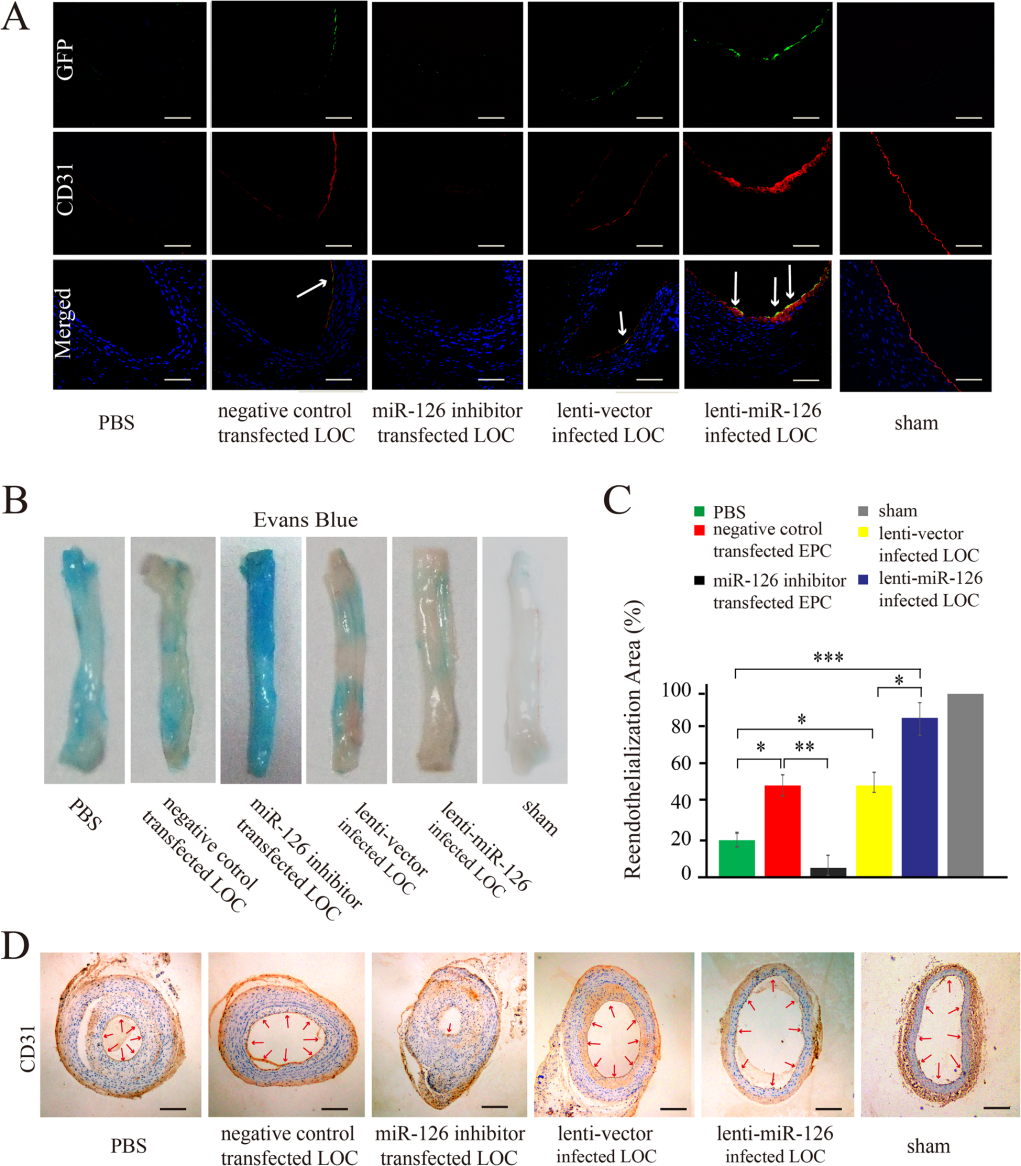
Effect of transplantation of lenti-miR-126-infected LOCs on reendotheliazation after carotid balloon injury in GK rats. Representative CD31 stained (red) cross sections of the injured carotid artery at day 7 after balloon injury in GK rats injected with PBS, or LOCs transfected with lenti-vector, or lenti-miR-126, or negative control or miR-126 inhibitor (**a**, × 200). Merged parts represent modified LOCs which are indicted by white arrows (× 200). Evans blue staining of injured carotid arteries (**b**) and quantification of reendotheliazation area ratio from Evans blue staining (**c**). CD31 immunohistochemical staining of carotid arteries from the above different groups at 28th day after injury (**d**, × 100). Red arrows indicate CD31 positive. *P < 0.05, **P < 0.005, ***P < 0.001, mean ± SD, *n* = 4. Scale bar = 100 μm

To induce endothelial damage, carotid artery balloon injury was performed in GK rats. Two hours after injury, either isovolumetric PBS or cell suspensions containing 1 × 10^6^ LOCs transfected with lenti-miR-126 or miR-126 inhibitor or their respective control were injected into tail veins of rats. At day 7 after injection, reendothelialization levels were examined by immunofluorescent staining of the endothelial marker CD31 (Fig. 8a) and Evans blue staining (Fig. 8b). Compared with PBS control group, transplantation of vector-infected LOCs or negative control transfected LOCs increased reendothelialization area (Fig. 8b, c), indicating that LOCs incorporated into injured vessels and improved reendothelialization after arterial injury. Transplantation of LOCs infected with lenti-miR-126 further enhanced CD31 immunofluorescence (Fig. 8a) and improved reendothelialization area on the top of the effect of lenti-vector infected LOC therapy (Fig. 8b, c). CD31 immunohistochemical staining at 28th day after injection indicated full reendothelialization in all groups except the group receiving miR-126 inhibitor-transfected LOCs (Fig. 8d).

#### Lenti-miR-126 infected LOCs decreased intimal hyperplasia after carotid artery injury

**Fig. 9:**
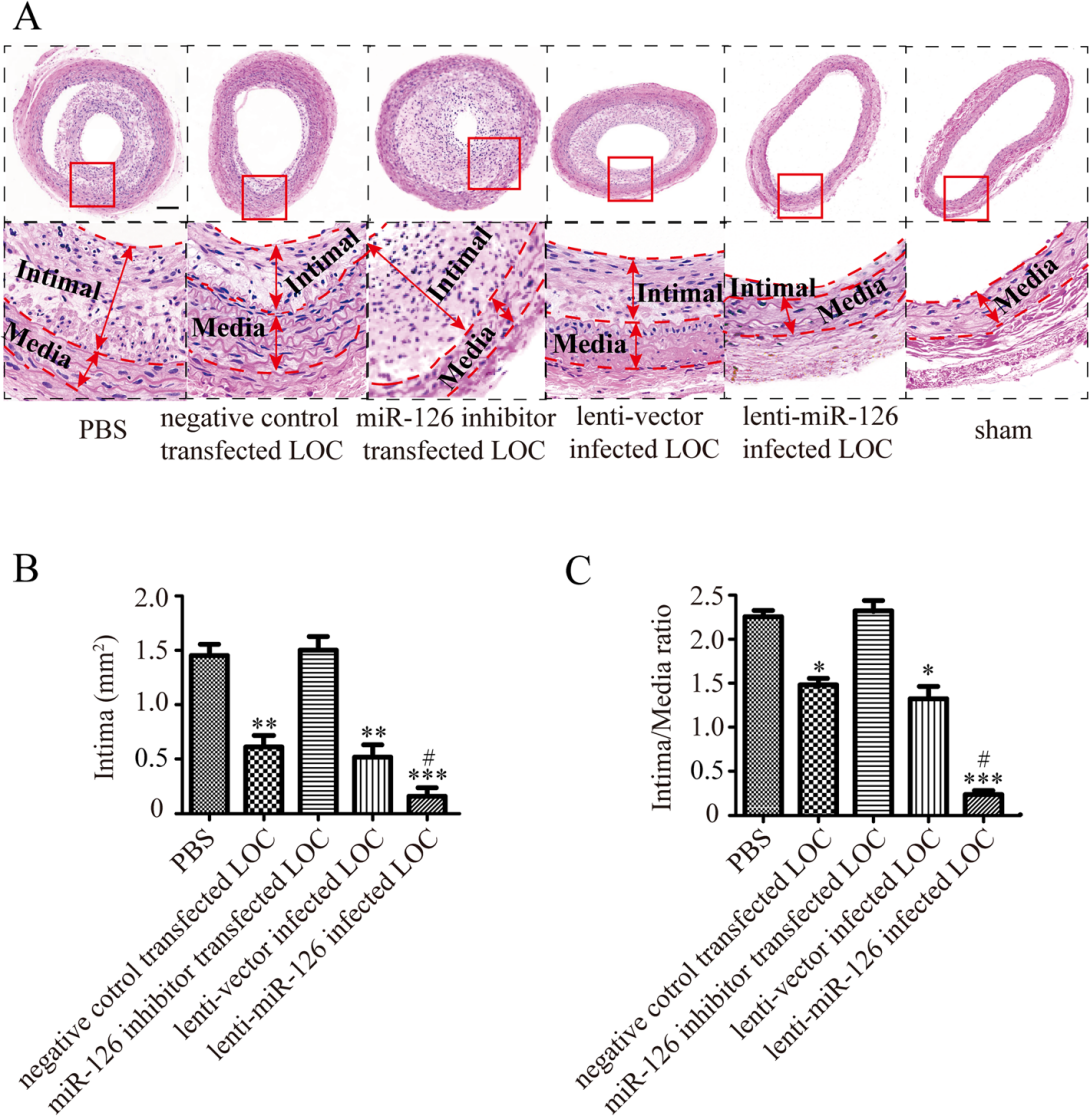
Effects of transplantation of lenti-miR-126 infected LOCs on neointimal hyperplasia in injured carotid arteries. HE staining of injured arteries 28 days after balloon injury in GK rats injected with PBS, or LOCs transfected with lenti-miR-126, or miR-126 inhibitor or their respective control or sham group (**a**, × 40), and red boxed areas are shown in panels below (**a**, × 400). Lines were used to indicate where the intima is and where the media is. Quantification of intima area (**b**) and intima/media ratios (**c**). *P < 0.05, **P < 0.01, ***P < 0.001 vs. PBS control; #*P* < 0.05 vs. lenti-vector infected LOCs; mean ± SD, *n* = 4. Scale bar = 100 μm

Last, we assessed the effects of different treatments on the extent of arterial intimal hyperplasia in carotid artery on 28th days after balloon injury in GK rat. Compared with the PBS control group, there was a reduction in both carotid intimal area and intima/media ratio in the GK rats injected with vector-infected LOCs or negative control transfected LOCs (Fig. 9a–c), indicating LOC injection reduced intimal hyperplasia after carotid artery injury. A further reduction in carotid intimal hyperplasia and intima/media ratio was noted in lenti-miR-126-infected LOC group compared to vector-infected LOC group (Fig. 9a–c), indicating that modulation of LOCs with overexpression of miR-126 has greater therapeutic potential in vascular injury.

## Discussion

The homing of EPCs into injured endothelium and ischemic tissues is a key step for the EPCs to exert protective effects on vascular injury and ischemia of organs, which is mediated by chemokines on the site of vascular injury and their receptors expressed on the surface of EPCs. Among all chemokine signaling pathways, SDF-1α/CXCR4 axis plays a pivotal role in EPC migration and homing [24, 39, 40]. The chemokine receptor CXCR4 belongs to the large family of G protein-coupled receptors, and EPCs incubated with CXCR4 antibodies or EPCs from CXCR4+/− mice displayed an impaired incorporation of EPCs into the sites of ischemia-induced neovascularization [41]. CXCR4 expression was significantly reduced on EPCs from patients with diabetes or on EPCs treated by high glucose [25], which could contribute to dysfunctional EPCs in diabetes. miR-126 was reduced in diabetic EPCs [23] and also in circulation, endothelial microparticles, and microvessicles in diabetic patients [42–44], which may contribute to impaired endothelial repairing capacity [23]. The above findings led to our hypothesis that miR-126 may mediate EPC mobilization via regulation on CXCR4. Indeed, in the present study, we demonstrated that overexpression of miR-126 significantly upregulated while inhibition of miR-126 significantly downregulated CXCR4 expression on LOCs, confirming that miR-126 promotes LOC migration via upregulating CXCR4 expression on LOCs. miRNAs are well-known negative regulators of gene expressions, but miRNAs have also been shown to target promoter sequences and induce gene expression [45].

Furthermore, we found that miR-126 increased p-ERK, VEGF, p-Akt, and eNOS protein expression and inhibitors of these proteins not only blocked miR-126-induced CXCR4 expression, but also reduced miR-126-induced migration in the presence of SDF, suggesting that the effects of miR-126 on CXCR4 and EPC migration are mediated by ERK/VEGF and Akt/eNOS signaling pathways. The roles of the above signaling molecules in regulating CXCR4 expression and EPC function have been demonstrated previously. Augmented CXCR4 expression in EPCs exposed to hypoxia was regulated by the PI3K/Akt signal transduction pathway [46]. Nitric oxide donor also promoted bone marrow stromal cell migration into ischemic brain through increasing endogenous SDF-1 and CXCR4 expression [47], while PD98059, an inhibitor of p-ERK, inhibited EPC proliferation and migration [48]. In addition, since PI3K/Akt is the upstream signaling pathway that regulates miR-126 expression in EPCs [49], the interaction between miR-126 and PI3K/Akt regulates CXCR4 expression on LOCs in a positive feedback loop. Taken together, in diabetic patients, high glucose downregulates miR-126 in EPCs [49], which, in turn, downregulates CXCR4 expression and inhibits SDF-1α mediated EPC mobilization via ERK/VEGF and Akt/eNOS signal pathways, subsequently impairing endothelial repairing ability of EPCs in diabetic patients.

Stemness maintenance of stem cells is important for the regeneration of ischemic myocardium [26]. Dawn et al. reported that CXCR4-SSEA-Oct4+stem cells, a kind of self-renewal proliferating cells, played an active role in tissue repair following myocardial infarction [44]. In the present study, we found that anti-miR-126 significantly decreased while overexpression of miR-126 significantly increased gene and protein expression of stemness markers in LOCs. Adrienne et al. also found that miR-126 contributed to the stemness maintenance of leukemia stem cells [27]. KLF family members play critical roles in vascular wall homeostasis and maintaining stemness [28]. The KLF family has been shown to play an important role in maintaining stemness [29, 30]. KLF-9 inhibited the stemness of glioblastoma cells [30]. KLF-2 contributed to the stemness maintenance and self-division of human mesenchymal stem cells [29]. We further demonstrated that KLF-8 was a direct target of miR-126 and that KLF-8 inhibited the stemness of LOCs, which abrogated the effect of miR-126 on promoting the stemness of LOCs.

EPC therapy represents a novel strategy for a variety of disease. Previous studies have shown that transplantation of EPCs promotes revascularization and blood flow recovery in ischemic organs including heart, brain, and limbs [50–52]. However, circulating EPCs are reduced and dysfunctional in diabetes and cardiovascular disease [16, 18, 53], so favorably modulating EPC function is critical for the therapeutic success in autologous EPC transplantation in these patients. In the present study, we modulated miR-126 expression in LOCs obtained from bone marrow of Wistar rats and then injected them into tail veins of GK rats 2 h after carotid artery balloon injury. First, we found that transplantation of LOCs infected with vector alone improved reendothelialization at 7 days and reduced intimal hyperplasia at 28 days after injury, confirming that injection of healthy LOCs protects against arterial injury in GK rats. Then we found that overexpression of miR-126 in LOCs promoted the homing of LOCs into the site of injury. Compared to LOCs infected with vector alone, LOCs infected with miR-126 resulted in significant improvement in their repairing ability in injured carotid arteries. In contrast, injection of LOCs transfected with miR-126 inhibitor exerted deleterious effects on arterial injury at 28 days after injury. Consistently, Bijkerk et al. demonstrated that overexpression of miR-126 in total bone marrow increased the number of vasculogenic progenitor cells and led to a marked protection of the kidney vasculature following ischemia reperfusion injury [54]. In addition, they found that overexpression of miR-126 in bone marrow induced renal epithelial SDF-1 expression. It has been shown that miR-126 modulates SDF-1 expression directly [55] and indirectly via repressing the function of regulator of G protein signaling 16 (RGS16). Silencing of RGS16 enables CXCR4 to trigger an autoregulatory feedback loop that increases the production of SDF-1 [56]. As such, they function in a coordinated network regulating VEGF signaling in endothelial cell quiescence and reendothelialization in endothelial injury or stress. Wu et al. demonstrated that EPC-derived microvesicles of diabetic patients carried less miR-126 and transferring of miR-126 via microvesicles enhanced the function of EPCs [43]. Chen et al. showed that the CXCR4 level was decreased in EPCs from diabetic mice and transfusion of CXCR4-primed EPCs reduced cerebral ischemia damage and promoted repair in db/db diabetic mice [57]. Our current study has proposed a direct regulatory effect of miR-126 on CXCR4 expression and its signaling pathways. The role for miR-126 in mediating EPC homing and improving vascular repair is successfully demonstrated with transplantation of miR-126 modified EPCs in GK rats. What is more, we found that miR-126 increased stemness via negatively regulating KLF-8 expression. Thus, our findings link miR-126 and homing and stemness maintenance in regulating EPC function, which adds important information to better understand the role of miR-126 in improving endothelial repair. Taken together, modulating EPCs with overexpressing miR-126 could improve reendothelialization and endothelial repair of vascular injury via promoting homing of LOCs with increased stemness into the injury site in diabetes and other diseases.

## Conclusion

In conclusion, miR-126 promotes LOC mobilization via upregulating CXCR4 expression, which is mediated by ERK/VEGF and Akt/eNOS pathways, and contributes to stemness maintenance by downregulating KLF-8. Transplantation of LOCs with overexpression of miR-126 improves reendothelialization and vascular repair after carotid artery balloon injury in a non-obese diabetes rat  model. Thus, miR-126 may be a new therapeutic target to reduce cardiovascular risk in patients with diabetes and cardiovascular disease.

## Supplementary information

Additional file 1: **Figure S1.** Characterization of LOCs derived from peripheral blood. LOCs with spindle shape formed clones (A, 100×; B, 200×). Isolated LOCs were GFP positive from GFP positive Wistar rats (Green) (C, 200×). Most LOCs were shown to simultaneously endocytose DiI-ac-LDL (red) (D, 100×) and bind fluorescein isothiocyanate UEA-1 (lectin, green) (E, 100×) from normal Wistar rats. Merged photo of 1D and 1E was also presented (F, 100×). FACS analysis showed high expressions of CD133, CD34 and KDR, and low expression of CD31 (G, mean ± SD) in LOCs. Scale bar = 100 μm. (TIF 4576 kb)

Additional file 2: **Figure S2.** Effect of KLF-8 on the migratory of LOCs. Crystal violet staining was performed to determine the number of migrated cells (A). Cell counts per high-power field were analyzed by image J (B). Data are presented as mean ± SD. *P < 0.05, **P < 0.01 and ***P < 0.001, vs respective control group; *n* ≥ 3. Scale bar = 200 μm. (TIF 3036 kb)

Additional file 3: **Figure S3.** Effect of miR-126 on the stemness of LOCs. Immunofluorescence was used to confirm CXCR4 expression on the cell membrane of LOCs in different groups. Scale bar = 50 μm. (TIF 4431 kb)

## Abbreviations

AGEs: Advanced glycation end products
BMMNCs: Bone marrow-derived mononuclear cells
CXCR4: Chemokine receptor 4
EPCs: Endothelial progenitor cells
FBG: Fasting blood glucose
LDL-ac-Dil: 1,19-Dioctadecyl-3,3,3939-tetramethylindocar-bocyanine perchlorate-labeled acetylated low-density lipoprotein
LOCs: Late outgrowth EPCs
IL: Interleukin
SDF-1α: Stromal cell-derived-factor 1α
TNF-α: Tumor-necrosis factor-α
miR: MicroRNA
FITC-UEA-1: Fluorescein isothiocyanate conjugated lectin Ulex europeus agglutinin 1
