# Supplementary figures and images for: MicroRNA-126 protects against vascular injury by promoting homing and maintaining stemness of late outgrowth endothelial progenitor cells
Source: Stem Cell Res Ther. 2020 Jan 21;11:28. doi: 10.1186/s13287-020-1554-9 (PMC6975061; doi:10.1186/s13287-020-1554-9)

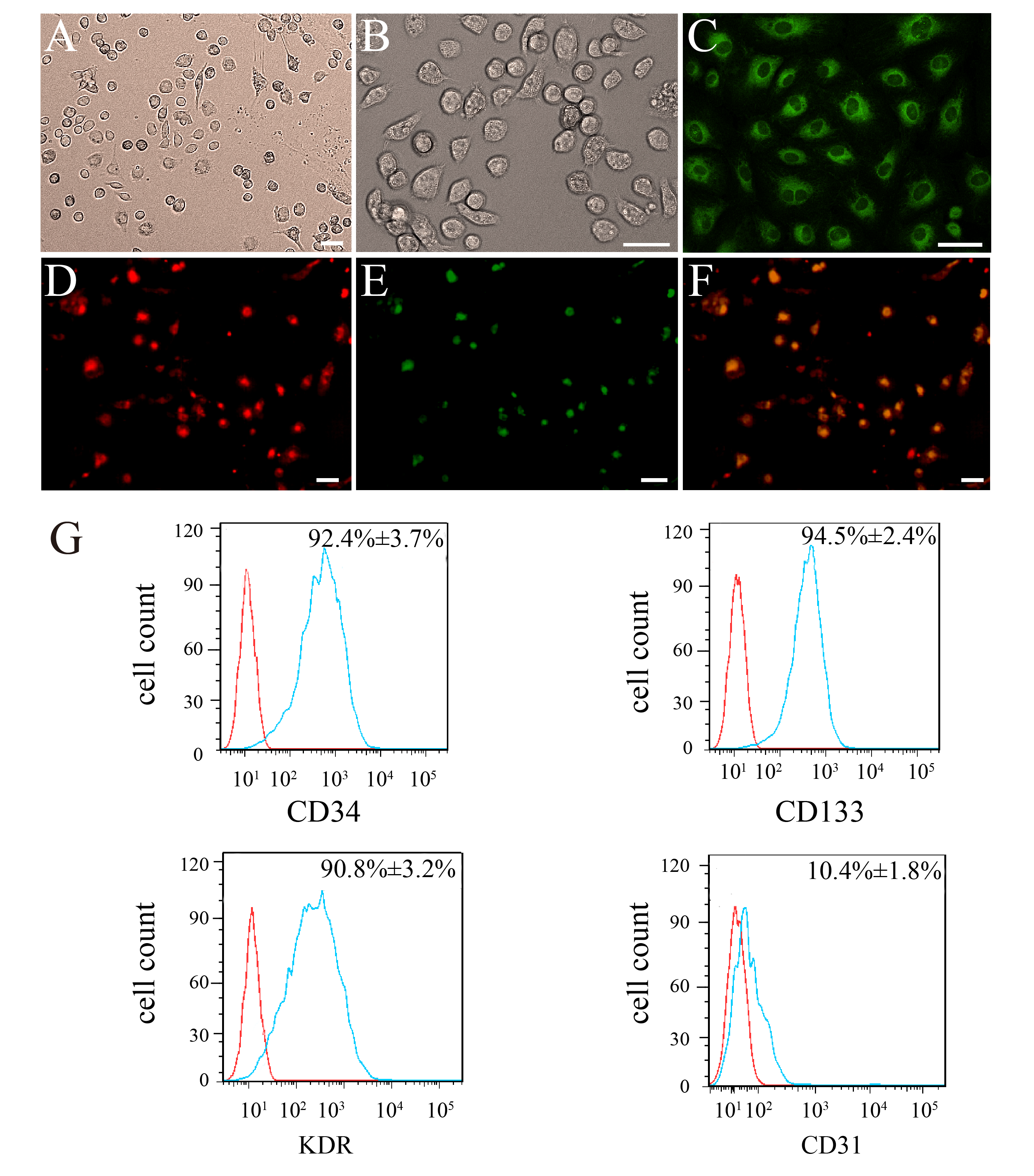

Supplement: Supplementary file 1 — Figure S1. Characterization of LOCs derived from peripheral blood. LOCs with spindle shape formed clones (A, 100×; B, 200×). Isolated LOCs were GFP positive from GFP positive Wistar rats (Green) (C, 200×). Most LOCs were shown to simultaneously endocytose DiI-ac-LDL (red) (D, 100×) and bind fluorescein isothiocyanate UEA-1 (lectin, green) (E, 100×) from normal Wistar rats. Merged photo of 1D and 1E was also presented (F, 100×). FACS analysis showed high expressions of CD133, CD34 and KDR, and low expression of CD31 (G, mean ± SD) in LOCs. Scale bar = 100 μm. (TIF 4576 kb) [file 13287_2020_1554_MOESM1_ESM.tif]

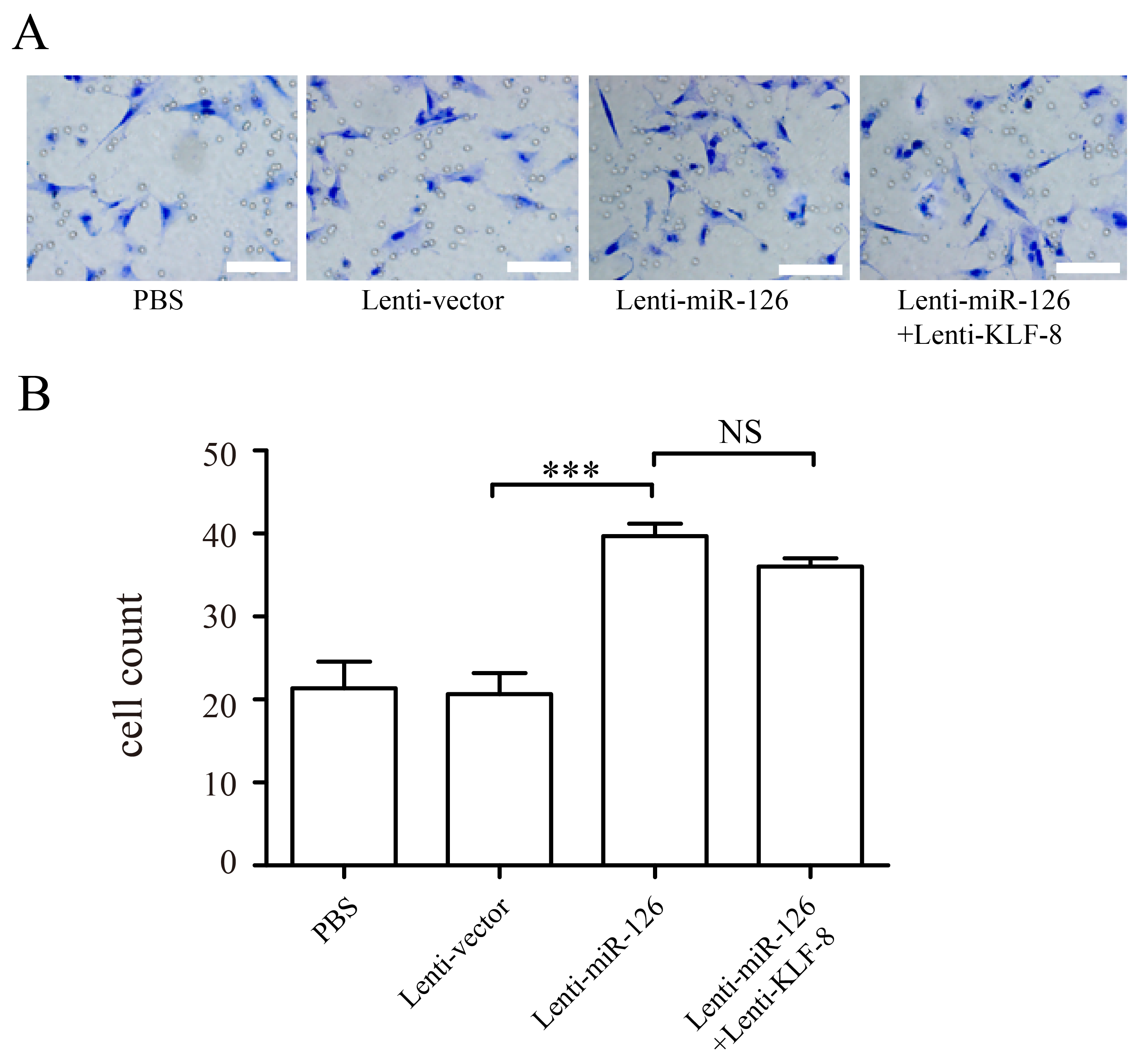

Supplement: Supplementary file 2 — Figure S2. Effect of KLF-8 on the migratory of LOCs. Crystal violet staining was performed to determine the number of migrated cells (A). Cell counts per high-power field were analyzed by image J (B). Data are presented as mean ± SD. *P < 0.05, **P < 0.01 and ***P < 0.001, vs respective control group; n ≥ 3. Scale bar = 200 μm. (TIF 3036 kb) [file 13287_2020_1554_MOESM2_ESM.tif]

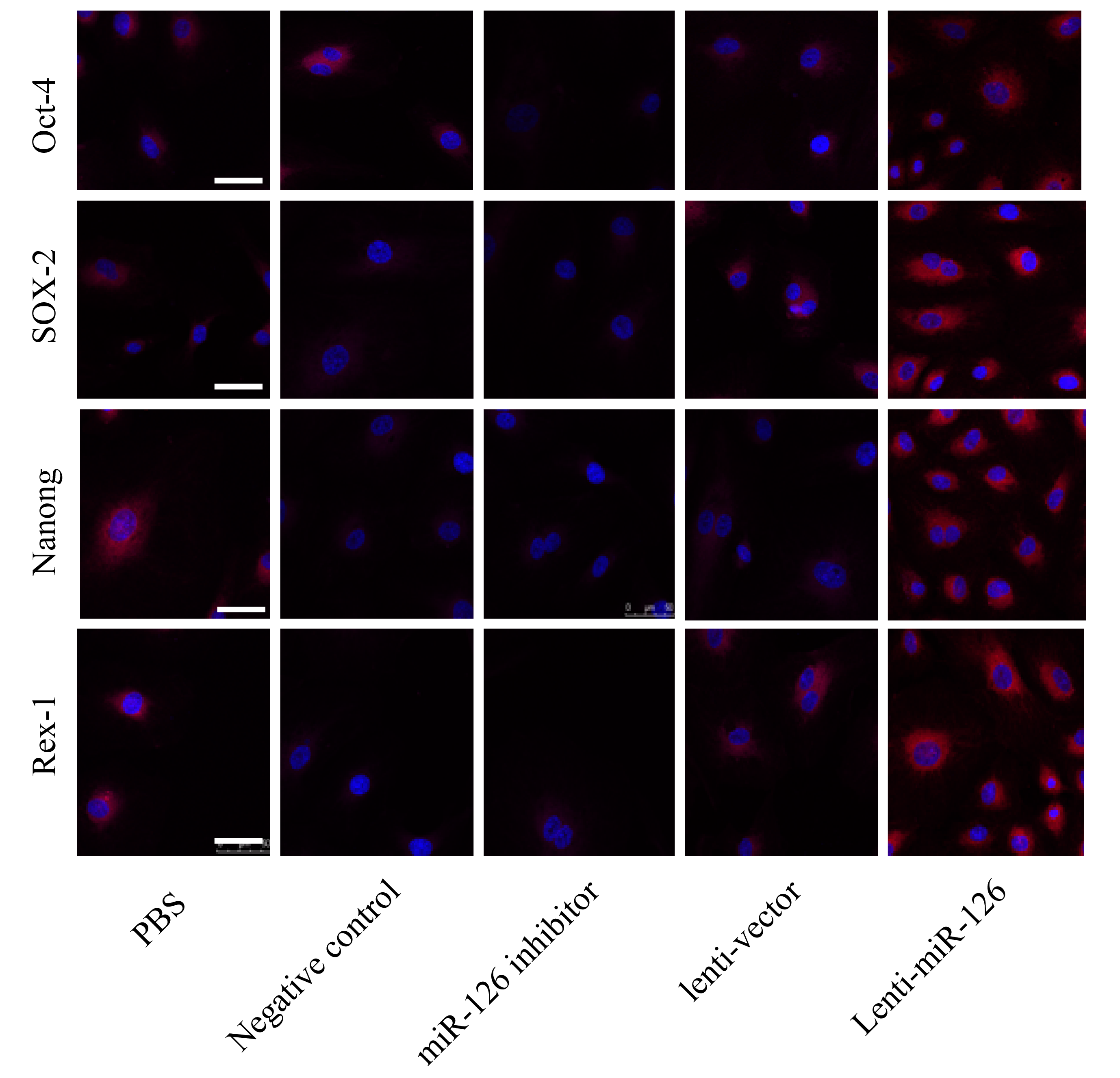

Supplement: Supplementary file 3 — Figure S3. Effect of miR-126 on the stemness of LOCs. Immunofluorescence was used to confirm CXCR4 expression on the cell membrane of LOCs in different groups. Scale bar = 50 μm. (TIF 4431 kb) [file 13287_2020_1554_MOESM3_ESM.tif]
